# Genetic and metabolic engineering approaches for enhanced biodesulfurization of petroleum fractions

**DOI:** 10.3389/fbioe.2024.1482270

**Published:** 2024-10-28

**Authors:** Asheemita Bagchi, Preeti Srivastava

**Affiliations:** Department of Biochemical Engineering and Biotechnology, Indian Institute of Technology Delhi, New Delhi, India

**Keywords:** desulfurization, biodesulfurization, mutagenesis, surface display, 4S pathway, Dsz enzymes, random chimeragenesis on transient templates

## Abstract

Sulfur, an abundant component of crude oil, causes severe damage to the environment, poses risks to human health, and poisons the catalysts used in combustion engines. Hydrodesulfurization, the conventionally used method, is not sufficient to remove thiophenes like dibenzothiophene (DBT) and other aromatic heterocyclic compounds. The push for “ultra-clean” fuels, with sulfur content less than 15 ppm, drives the need for deep desulfurization. Thus, in conjunction with hydrodesulfurization, efficient and eco-friendly methods of deep desulfurization, like biodesulfurization, are desirable. In biodesulfurization, naturally desulfurizing microorganisms are used, with genetic engineering and biotechnology, to reduce the sulfur content of crude oil to below 15 ppm. In this review, we describe genetic and metabolic engineering approaches reported to date to develop more efficient methods to carry out biodesulfurization, making it a practically applicable reality.

## Introduction

Sulfur emissions due to fossil fuel combustion are a global problem. To reduce the sulfur content, several processes such as oxidative desulfurization, ionic desulfurization, adsorptive desulfurization, hydrodesulfurization, and biodesulfurization are used (Nazir et al., 2021). Biodesulfurization involves the use of microbes for the removal of sulfur. A number of review articles describing the process have been published (Babich and Moulijn, 2003; Boniek et al., 2015; Gray et al., 2003; Kilbane II, 2006; Kropp and Fedorak, 1998; McFarland, 1999; Mohebali and Ball, 2016; Monticello, 2000; Nuhu, 2013; Ohshiro and Izumi, 1999; Sadare et al., 2017; Soleimani et al., 2007). The biodesulfurization rates of naturally occurring microorganisms are low, highlighting the need to improve the host organism using various biotechnological approaches. To make biodesulfurization a practically applicable reality, both biomolecular and bioprocess engineering approaches are required. In this paper, we describe various biomolecular engineering approaches, including genetic and metabolic engineering, that have been reported for improving biodesulfurization and discuss their practical applicability.

### Sulfur in crude oil

Sulfur, in its various forms, is an abundant component of crude oil. Its abundance varies from 0.05% to 10%, and it can be present in its elemental form or in the form of sulfide, sulfate, and sulfite and has more than 200 organic forms. Sulfur-containing heterocyclic organic molecules are very notorious environmental pollutants (Mohebali and Ball, 2016).

Crude oil contains several categories of sulfur-containing organic compounds: (i) aliphatic and aromatic thiols and their oxidation products (disulfides); (ii) aliphatic, aromatic, and mixed thioethers; and (iii) heterocyclic compounds with a thiophene ring, including thiophenes, benzothiophenes (BTs), dibenzothiophenes (DBTs), and their alkyl-substituted derivatives (Kropp and Fedorak, 1998). Approximately 50%–95% of the total amount of sulfur in crude oil is constituted of thiophenic sulfur. Among the organosulfur compounds in crude oil, the most common is DBT (Mohebali and Ball, 2016).

### Problems because of the sulfur in crude oil

The combustion of fossil fuels leads to the emission of NOx, SO_X_, and other particulate matter (PM) that later react with atmospheric oxygen and moisture, leading to a solution of very dilute sulfuric and nitric acids, which, upon precipitation, results in “acid rain.” Acid rain has destructive effects on the environment as it causes an imbalance in the natural chemical levels in the soil and also has an overall negative impact on the ecosystem. Moreover, the sulfur content in the diesel used in engines poisons the oxidation catalysts and reduces the effectivity needed to oxidize lethal components like CO, other hydrocarbons, and volatile compounds. The amount of PM obtained by the combustion of diesel is proportional to the amount of sulfur present in it, and PM is associated with disastrous effects on human health, like lung cancer and cardiopulmonary mortality (Stanislaus et al., 2010).

### What is desulfurization?

Desulfurization can be broadly defined as the process of reducing the level of sulfur in fuels to lessen its harmful environmental and economic impacts. Direct reductions in sulfur oxides and sulfur particulate matter can be achieved through three main ways, namely, optimizing and designing better performing emission systems, developing better, more efficient catalysts, and using and developing better filters or adsorbers to eliminate or lessen the particulate matter and several such oxide gases (Stanislaus et al., 2010).

### Conventional methods of desulfurization

Hydrodesulfurization (HDS) is the method used by industries and refineries to remove organic sulfur from fuels. The advantage of HDS is that it is efficient in removing a wide variety of sulfur compounds and not just organosulfurs. The more complex compounds like DBTs, BTs, and polyaromatic sulfur heterocyclic compounds are resistant to HDS (Mohebali and Ball, 2016).

Deep desulfurization is the process used to reduce sulfur content to approximately 15 ppm in crude oil to obtain ultra-clean fuels. This process demands more energy and leads to larger amounts of greenhouse gas emissions (Stanislaus et al., 2010).

### Biodesulfurization

Biodesulfurization (BDS) is the removal of sulfur using microorganisms. It can target the DBTs that are recalcitrant to HDS. It is cost-effective as it reduces the capital and operating costs of the process. It is also environmentally friendly. The technology to utilize the process in large-scale applications has not yet been developed and is still under research. Bacteria that utilize complex organosulfur compounds to break them down for their carbon skeleton or the sulfur moiety are abundantly found in various geographic environments (Mohebali and Ball, 2016). The most commonly used sulfur compound in BDS research is DBT and its derivatives because they form a large fraction of the most commonly used crude oils. As mentioned earlier, HDS cannot remove DBTs and their alkylated derivatives, so the potential of BDS to achieve this needs to be investigated (Oldfield et al., 1998). Recent reports have enlisted the different biodesulfurizing strains described to date (Al-Khazaali et al., 2023; Mamuad and Choi, 2023). Few newly isolated biodesulfurizing strains belonging to the phylum Actinobacteria have also been reported (Akram et al., 2024; Parveen et al., 2024).

Aerobically, DBT and its derivatives can be degraded by three pathways by microbes. The first is the Kodama pathway, where the carbon skeleton is partially oxidized and the bond between carbon and sulfur remains intact (Kodama et al., 1973). In the second pathway, DBT is used as the sole source of carbon, sulfur, and energy (Van Afferden et al., 1990). The third is the 4S pathway, where the compound is broken down to free the sulfur, but the carbon framework remains intact (Gallagher et al., 1993; Oldfield et al., 1997). According to another classification, there are two pathways: ring-destructive and sulfur-specific. The aerobic sulfur-specific pathway is the 4S pathway, where the overall calorific value of DBT remains unchanged as the carbon skeleton is not broken down. It is undertaken by four desulfurizing enzymes, namely, DszC, DszD, DszA, and DszB (Mohebali and Ball, 2016).

### Dsz enzymes

**FIGURE 1:**
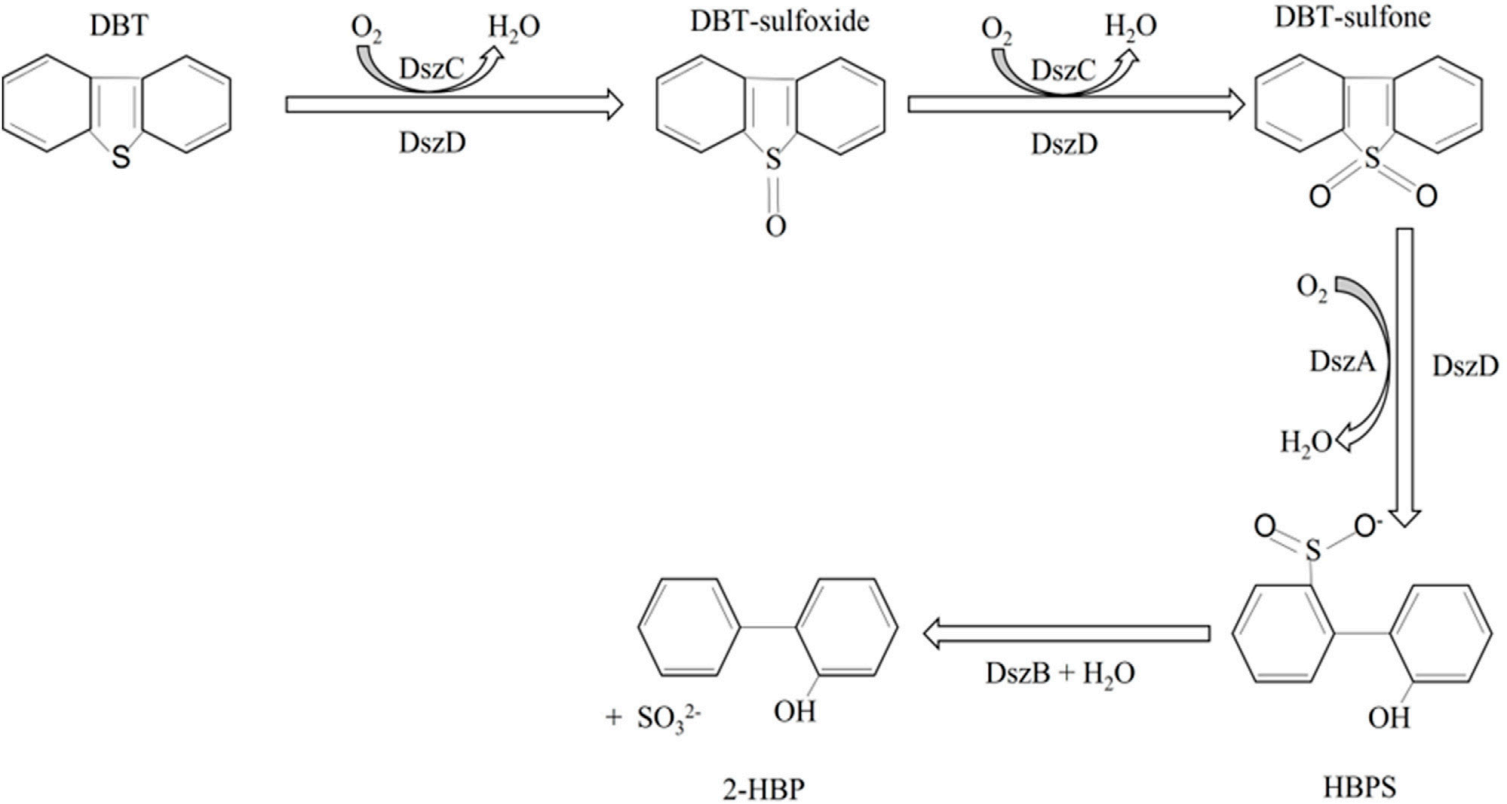
The 4S pathway of microbial desulfurization, dibenzothiophene (DBT), gets converted to DBT sulfoxide and DBT sulfone in two steps catalyzed by DszC (monooxygenase). DBT sulfone gets converted to 2-hydroxybiphenyl sulfite by the action of DszA (monooxygenase). DszD, a flavin oxidoreductase, is an auxiliary enzyme and is also involved in all the oxygenation steps. The last step is catalyzed by a desulfinase, DszB, yielding the final product, 2-hydroxybiphenyl. Adapted from Mohebali and Ball (2016).

The genes encoding the desulfurization enzymes are all part of the same *dsz* operon. They are a group of two monooxygenases (DszC and DszA) and a desulfinase (DszB). These three enzymes involved in the 4S pathway catalyze the conversion of DBT via a series of sequential reaction steps to yield 2-hydroxybiphenyl (2-HBP) and sulfite. DBT is first oxidized to DBT sulfoxide using DszC and DszD and then again oxidized to form DBT sulfone with the same set of enzymes. These two steps require molecular oxygen and FMNH_2_, the latter being supplied by NADH:FAD oxidoreductase (DszD). DBT sulfone is then converted to hydroxybiphenyl sulfite (HBPS), which is a sulfinate compound. This step is catalyzed by DszA, another monooxygenase, and DszD. HBPS is then acted on by a sulfinic acid hydrolase, DszB, to break down into 2-HBP and sulfite ions (Figure 1). The last step is the rate-limiting step of the 4S pathway. It should be noted that the 4S pathway is an energy-intensive process; approximately 4 moles of NADH are required to desulfurize 1 mole of DBT (Mohebali and Ball, 2016). Despite the wide range of knowledge available about the biodesulfurization pathways, especially the 4S pathway, efforts to scale it up to be applicable in an industrial setup have proven to be unsuccessful (Sar et al., 2021). Several developments have been made to overcome this drawback, some of which are discussed below.

### Biotechnological approaches

#### Improving the cellular growth and metabolism by co-culture and supplementing Vitreoscilla hemoglobin

*Vitreoscilla* is an obligate aerobic bacterium that is classified under purple bacteria. It produces Vitreoscilla hemoglobin (VHb), a large soluble dimeric protein, under hypoxic conditions (Lee et al., 2004). It was the first microbial hemoglobin to be discovered, as early as 1986. Cloning the gene for VHb and expressing it in a heterologous host has yielded striking results regarding cellular metabolism, the behavior of certain enzymes in the cell, and even the metabolism of certain compounds (aromatic) (Stark et al., 2011). Initially believed to be a cytochrome, albeit the soluble nature of the protein, the amino acid sequence confirmed it to be a hemoglobin (Khosla and Bailey, 1988; Wakabayashi et al., 1986).

**FIGURE 2:**
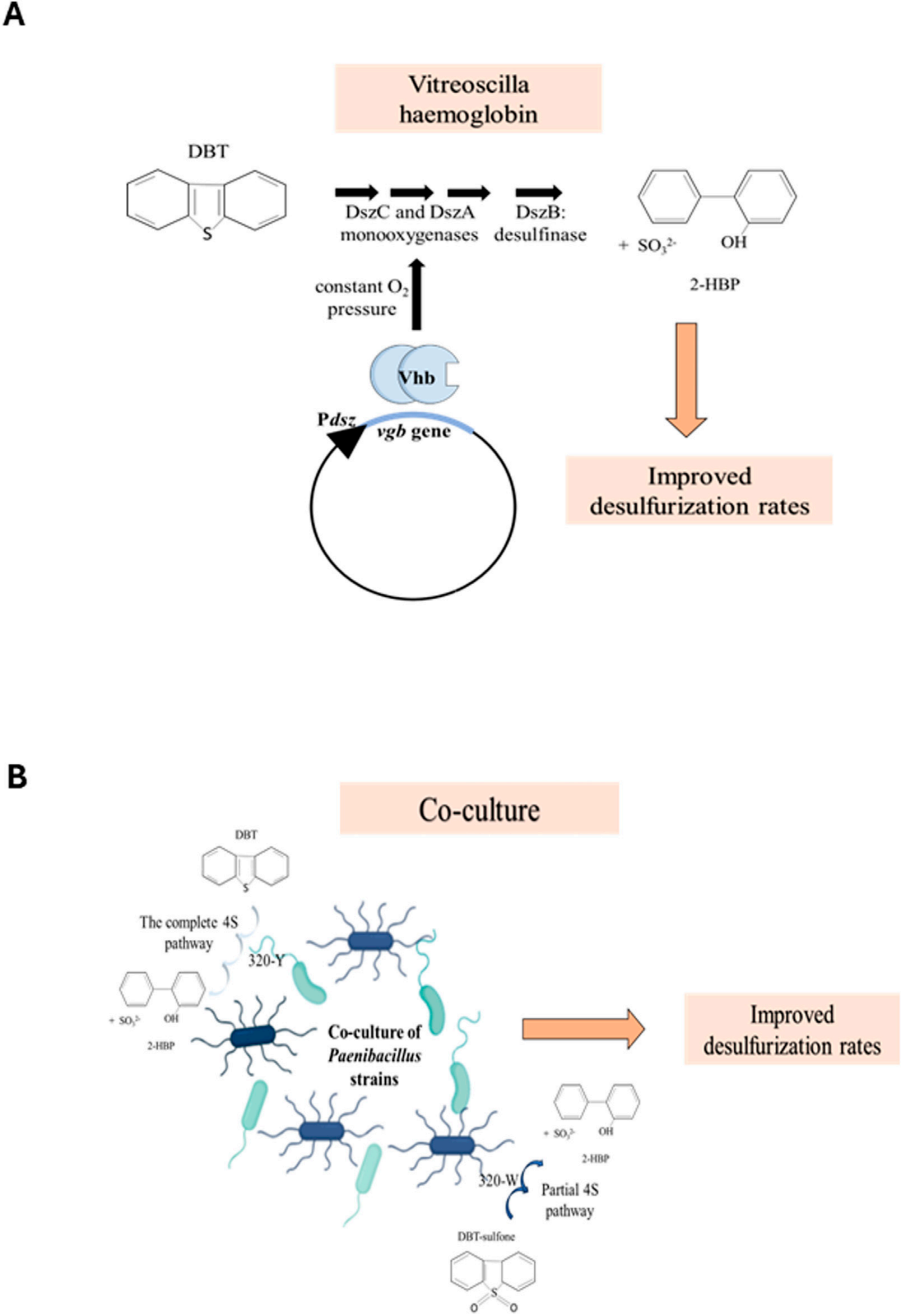
**(A)** The *vgb* gene, from *Vitreoscilla* sp., cloned under the native promoter, P*dsz*, scavenges and sequesters oxygen in the cytoplasm, thus maintaining a constant oxygen pressure for the DszC and DszA monooxygenases. This, in turn, increases the overall rate of the 4S pathway (adapted from Xiong et al. (2007)). **(B)** Co-culturing of the two strains of *Paenibacillus* sp., 320-Y and 320-W, increases the rate of desulfurization. 320-Y performs the complete 4S pathway, while 320-W metabolizes DBT sulfone to 2-HBP, an incomplete 4S pathway (adapted from Wang et al. (2015)).

The expression of the *vhb* gene in *Rhodococcus erythropolis* resulted in the increased removal of sulfur from diesel oil and DBT by 9%–38% (Stark et al., 2011; Xiong et al., 2007). The need for Vitreoscilla hemoglobin in desulfurization by biodesulfurizing strains stems from the fact that two enzymes in the 4S pathway, DszC and DszA, are monooxygenases; thus, the bacteria need not only oxygen supply for their own endogenous metabolism but also for desulfurization. However, the Michaelis constant (Km) of the enzymes for oxygen is high, thus requiring high oxygen pressure throughout the process (Xiong et al., 2007). The desulfurizing bacterium, *Rhodococcus erythropolis* LSSE8-1, was transformed with the *vgb* gene, cloned under the native *dsz* promoter, and referred to as LSSE8-1-vgb (Figure 2A). As hypothesized, the rate of desulfurization was significantly higher in the recombinant (37.5% in the case of DBT), LSSE8-1-vgb, than in the control cells (20.5%). Similar results were obtained with diesel oil. The benefits of having desulfurizing enzymes soluble in the cytoplasm, which aids in the transfer of oxygen to the monooxygenases by VHb, are also noted. As a result, there was a significant improvement in desulfurizing activity due to VHb, even in the absence of hypoxic conditions (Xiong et al., 2007).

Several groups reported that the co-culture of different desulfurizing bacteria or a mixed culture has a synergistic effect, yielding better growth and increased biodesulfurization (Martínez et al., 2016; Wang et al., 2015). Wang et al. reported two strains of *Paenibacillus*, 32O-Y and 32O-W, that could carry out the desulfurization of DBT and DBT sulfone (DBTS), respectively. The strain 32O-Y carried out the 4S pathway to desulfurize DBT, while 32O-W performs what appears to be a partial 4S pathway to metabolize an intermediate, DBT sulfone. Thus, the mixed culture of these two strains has a symbiotic relationship, leading to increased desulfurization by 32O-Y as opposed to when only 32O-Y was cultured (Figure 2B). To assess the effect of VHb, the *vgb* gene was transformed into the 32O-Y strain, and the transformed cell, now called 32O-Yvgb, was co-cultured with 32O-W. These were grown in varying concentrations of DBT and DBTS to study their desulfurization activities. The VHb was calculated to be 7.6 nmol/g of wet weight of cells, consistent with other reports on the heterologous expression of VHb in other cells (Sar et al., 2021). The co-culture of 32O-Yvgb and 32O-W showed the highest cell mass. However, contrary to what was hypothesized, all the experimental strains, including the co-cultures of 320-Yvgb and 320-W, showed similar trends in DBT degradation throughout the course of the experiment across all different initial DBT concentrations. Although it was concluded that the Vitreoscilla hemoglobin had a positive effect on biodesulfurization, there still needs to be ample research conducted on its applicability before extrapolating these results to industry (Sar et al., 2021). It was proposed that the enhanced growth of the co-culture of 32O-Y and 32O-W could be not only due to 32O-W utilizing DBTS of the 4S pathway but also due to sulfite produced by 32O-Y, thus driving the flux of the 4S pathway (Wang et al., 2015).

### Effect of the gene dosage, plasmid copy number, and sulfur sink

A shuttle vector was constructed between *Rhodococcus erythropolis* KA2-5-1 and *E. coli* using the cryptic plasmid pRC4 from *R. rhodochrous* IFO3338 (Hirasawa et al., 2001). The *dsz* operon having *dszA*, *dszB*, and *dszC* along with the reductase gene *dszD* was cloned from KA2-5-1, and the constructed plasmid was used to transform KA2-5-1. Thus, the transformant contained two clusters of *dszABC* and one *dszD*. The activity of the transformed organism was 4-fold higher than that of the parent strain (Hirasawa et al., 2001).

**FIGURE 3:**
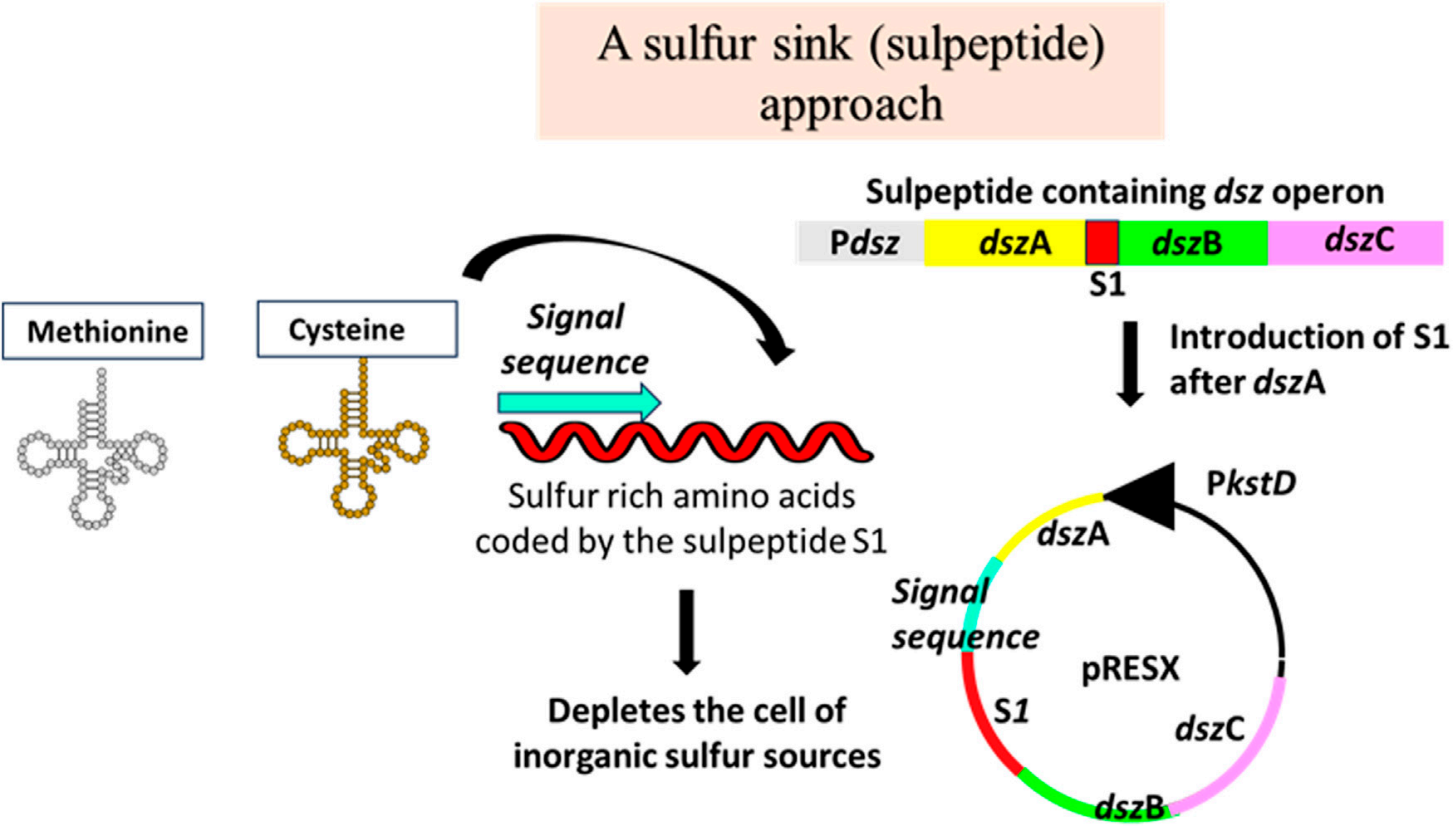
Sulfur sink: cloning a peptide high in sulfur-containing amino acids (S1) within the *dsz* operon ensures a near to complete depletion of inorganic sulfur sources in the cell, thereby pushing the cell to metabolize organic sulfur sources. Adapted from Pan et al. (2013).

A polypeptide rich in sulfur-containing amino acids, like cysteine and methionine, called sulpeptide, S1, was designed (Figure 3) (Pan et al., 2013). It was cloned between the genes *dsz*A and *dsz*B of a native *dsz* operon isolated from a desulfurizing strain of *Rhodococcus erythropolis*. This engineered operon was cloned under the P*kst*D promoter in a shuttle vector. The sulpeptide was a 267-bp-long sequence, having a leader sequence, a ribosome-binding site, a protein-coding region with a secretory signal sequence, and a 25-amino acid-long polypeptide having 13 sulfur-rich amino acids. The importance of the sulpeptide is that it provides the host bacterium with the pressure to keep expressing the *dsz* operon in which it is coded. The preferred sulfur sources for the bacteria are sulfates, cysteine, and methionine-containing compounds. By ensuring that the sulpeptide gets transported outside the cell, Pan et al. treated it as a sulfur source for the cell to catabolize. So, the drive to utilize the sulpeptide ensures that along with it, the *dsz* operon also gets expressed and the enzymes get produced by the cell. The sulpeptide essentially causes the complete depletion of all the other preferred sulfur sources in the cell. As a result, when the cell is grown in a medium containing DBT, the *dsz* operon that gets expressed helps the cell utilize the DBT via the 4S pathway. They worked with the WT *Rhodococcus erythropolis* IGTS8 and *Rhodococcus opacus* cells. Plasmids containing the native *dsz* operon, or the *dsz*A-S1-*dsz*BC, were electroporated. It was observed that the cultures that harbored the engineered operon, i.e., *dsz*A-S1-*dsz*BC, seemed to show greater desulfurization activity per subculture. After 40 subcultures, they noted a 23-fold and 35-fold increase in the *R. opacus dsz*ABC and *R. opacus dsz*A-S1-*dsz*BC, respectively. For the biodesulfurization process to become industrially suitable, the specific activity should be from 1,200 to 3,000 μmol of 2-HBP/g of dry cell weight/h. The average specific activity obtained from this work was sufficient because only a 10-fold improvement combined with the cumulative effects of process engineering could yield the desired amount needed for an industry-scale operation (Pan et al., 2013).

The same research group that came up with the sulpeptide investigated the idea of adaptive selection and directed evolution (Wang et al., 2017). An interesting discovery was that the sulpeptide, which was previously thought to be instrumental in adaptive selection, thereby increasing the rates of desulfurization, was only increasing the growth rate of the host and acting as a sulfur sink in the case of scarcity of other sulfur sources. Three separate selection experiments were designed to vary the carbon source and sulfate source using different strains. The desulfurization (specific activities measured in micromoles 2-HBP/g DCW/h) peaked during the fourth passage, then decreased, and stabilized at 23 units (micromole 2-HBP/g DCW/h-specific activity) for the strain with *dszABC* and 48 units for the strain with the sulpeptide. The increase in desulfurization in the strains was hypothesized to be due to better enzyme efficiency because of some mutations in the *dsz* genes. However, whole-genome sequencing disproved the hypothesis because no such mutations were detected. However, what was seen was an increase in the copy number of the plasmid carrying the *dsz* genes. Although the sulpeptide was previously shown to influence the derepressing of the *dsz* operon, interestingly enough, Wang et al. noted that the sulpeptide failed to provide a significant growth or desulfurization advantage to its host compared to the other strain carrying the native *dsz* operon (Pan et al., 2013; Wang et al., 2017). This could possibly be due to the sulpeptide supplying a rather small fraction of sulfur, which is not considerably sufficient to provide any sort of sulfur sink function to its host. So, the increase in the plasmid copy number was the only viable reason behind the increase in desulfurization in both the strains (Wang et al., 2017).

Overall, Wang et al. (2017) successfully engineered a non-desulfurizing strain called CW25 of *Rhodococcus qingshengii* to express a sulpeptide and carried out repeated passages (thus being aided by adaptive selection and directed evolution) in DBT-containing media. As a result, they noted a considerable increment in the desulfurization activity (DBT metabolism) and growth rate in the DBT-containing media (Wang et al., 2017). This particular experimental strategy can be exploited in the future to further elucidate the mechanism behind the results shown and also improve desulfurization.

### Co-expression of molecular chaperones

The DszB from the naturally desulfurizing organism, *Rhodococcus erythropolis* KA2-5-1, was purified to homogeneity and characterized in detail (Nakayama et al., 2002). The researchers have also developed a way of overexpressing the enzyme using molecular chaperones in *E. coli*, GroES/GroEL, which are credited for helping in the folding of many proteins in the bacteria. The *dszB* gene was amplified from the strain KA2-5-1, cloned in a pET21-a (T7 promoter) vector, and transformed to *E. coli*. The protein failed to be active, which was thought to be due to its presence in insoluble inclusion bodies in the organism. Even after the lowering of the temperature of growth to 25°C from 37°C, the activity was not high. With a different promoter, however (*tac* and *lac*), the activity of DszB was increased, and with the *tac* promoter, the activity was higher. Then, chaperone proteins were co-expressed along with the *dszB* gene. With GroES/GroEL at 25°C, the solubility and activity of DszB were significantly higher in the recombinant strain than in the WT KA2-5-1 strain. Thus, to avert the problem of the rate-limiting step of DszB, overproducing the enzyme in a soluble form by the co-expression of molecular chaperones can be a very potent solution (Nakayama et al., 2002).

### Flavin reductase from the heterologous host

Reichmuth et al. (2000) successfully improved the rates of desulfurization in recombinant *E. coli* expressing the *dszABC* genes and a flavin oxidoreductase gene from *Vibrio harveyi. E. coli* DH10B was transformed with a vector coding for the flavin oxidoreductase from *V. harveyi* with the lac P/O system, which can thus be tightly regulated with IPTG (Reichmuth et al., 2000). Desulfurization assays were performed with *E. coli* cultures harboring pDSR3 (having the *dsz* operon cloned under the P_BAD_ promoter). As the native *dsz* operon is sulfate-repressible, placing it under the arabinose inducible promoter solved the issue. The culture was easily grown in LB broth, which contains various sulfate sources. It also showed a significant amount of desulfurization as observed by depletion of the initial amount of DBT in the culture.

The resting cell system having *E. coli* harboring both pDSR2 and pDSR3 plasmids was then checked for desulfurization activity. After just 4 h, there was no measurable DBT in the solution, proving that the strain was efficient in desulfurization. However, on an interesting note, there seemed to be no generation of 2-HBP, the end product of the 4S pathway. It was theorized that even though heterologous oxidoreductase helped in enhancing the monooxygenase activities, it placed a metabolic burden on DszB, resulting in its low activity. This publication impressed upon the usage of a resting cell system better suited for industrial biodesulfurization (Reichmuth et al., 2000).

Galán et al. (2000) experimented with the same idea, using flavin reductase from *E. coli* W, called HpaC FMN:NADH oxidoreductase (Galán et al., 2000). Since resting-cell systems were better biocatalysts, the researchers proceeded to prepare recombinant *Pseudomonas* strains. The *in vitro* effect of HpaC reductase was investigated using soluble cell-free extracts containing DszA, DszC, and DszB, along with HpaC. There was a significant increase in the conversion of DBT to 2-HBP. A *Pseudomonas* strain with *hpa*C and *dszABC* genes integrated into their chromosome was used to create a resting-cell system. DBT degradation was achieved by the system, and in contrast to the findings obtained by Reichmuth et al. (2000), it was shown that expression of this oxidoreductase did not decrease in the formation of 2-HBP along with DBT depletion. Galán et al. then proceeded to develop a mobile DNA cassette having the genes *hpa*C and *dszABC* under the control of a P*tac* promoter, which was then used to transform various other Gram-negative strains that carried out desulfurization of DBT as well (Galán et al., 2000).

Both the above studies evidently demonstrated the importance of using a resting-cell system of engineered desulfurizing strains that have the potential to help make biodesulfurization a commercially established industrial process.

In the need to find a flavin oxidoreductase from a non-desulfurizing organism that couples with the monooxygenases DszA and DszC of *R. erythropolis* D-1, *Paenibacillus polymyxa* A-1 was found to have a flavin reductase that coupled efficiently (Ohshiro et al., 2002).

Around a hundred microbes were screened for the presence of such an enzyme. The flavin reductase, DszD of *R. erythropolis* D1, had more affinity toward NADH than NADPH. Closest to this trend was the flavin reductase from *P. polymyxa* A-1. The purified enzyme from *P. polymyxa* A-1 had a better affinity for FMN than for NADH. The enzyme was used to measure its coupling efficiencies with DszC with a wide range of flavin compounds as the electron acceptor and NADH or NADPH as donors. The enzyme showed better coupling with DszC of *R. erythropolis* D1 in the presence of NADH and FMN. *V. harveyi* also has a flavin reductase that has been researched regarding its coupling with the DszC of *R. erythropolis* D1. The enzyme from *P. polymyxa* A-1 is even better at coupling with DszC from D1 than that of *V. harveyi* (Ohshiro et al., 2002).

Ishii et al. (2000) reported a 10-fold increase in DBT desulfurization by a recombinant *E. coli* culture engineered to produce *dszABC* genes from *R. erythropolis* IGTS8 and the flavin reductase gene (*flv*) from *P. polymyxa* A-1 (Ishii et al., 2000). Primer walking was used to determine the sequence of the ORF of the flavin reductase. Through BLAST, it was seen that the sequence is homologous to similar reductases found in many organisms; however, it had no homology with that of rhodococcal DszD, probably reflected in the need for different cofactors by the two enzymes. The *flv* gene was cloned into a vector and transformed into *E. coli*, which had 40 times more oxidoreductase activity. The group then co-expressed the *dszABC* genes from *R. erythropolis* IGTS8 along with the *flv* gene. This culture showed 10-fold more desulfurization activity than did the strain having only *dsz*ABC*.* Another culture, containing the *dsz* operon from the thermophilic desulfurizing bacterium *Paenibacillus* sp. A11-2 and the *flv* gene, showed a 4.5-fold increase in activity (Ishii et al., 2000).

According to these findings, the flavin reductase from a non-desulfurizing organism was efficiently coupled with the monooxygenase of *Rhodococcus*, resulting in faster desulfurization (Ishii et al., 2000).

### Use of a non-repressible promoter

The *dsz* operon is repressed by inorganic sulfates and sulfur-containing amino acids like cysteine and methionine. In order to carry out an industrial process of biodesulfurization, the culture must be grown in a medium devoid of simple inorganic sulfur sources, and DBT must be the sole sulfur source. However, there are several concerns with this. The culture cells face sulfur starvation stress, lengthening the fermentation process, thus rendering the whole process extremely costly. DBT is also not homogenously mixed in the media, thus leading to an imbalance of sulfur resources among the cultures (Shavandi et al., 2009). So, several groups have worked on developing desulfurizing strains that have the *dsz* operon under the control of a sulfate non-repressible promoter. The non-repressible promoters used to date are described in Table 1.

**TABLE 1:** Heterologous promoters (which are not repressed by inorganic sulfur) used for expressing the *dsz* operon.

S. No.	Promoter	Source of the *dsz* operon	Expression host	Reference
1	P*BAD*	*Rhodococcus erythropolis* IGTS8	*Escherichia coli*	Reichmuth et al. (2000)
2	P*lac*	*Gordonia alkanivorans* RIPI90A	*Gordonia alkanivorans* RIPI90A	Shavandi et al. (2009)
3	P*hsp60*	*Mycobacterium* sp. G3	*Mycobacterium* sp. G3	Takada et al. (2005)
4	P*rrn*	*Rhodococcus* sp. T09	*Rhodococcus* sp. T09	Matsui et al. (2002)
5	P*neo*	*Arthrobacter* sp. DS-7	*Escherichia coli* TG1	Serbolisca et al. (1999)
6	P*com8*	*Rhodococcus* sp. FUM94	*Escherichia coli* BL21 (DE3)	Khosravinia et al. (2018)
7	P*spac*	*Rhodococcus erythropolis* DS-3	*Bacillus subtilis*	Ma et al. (2006)
8	P*kap1*	*R. erythropolis* KA2-5-1	*R. erythropolis* KA2-5-1	Noda et al. (2002)
9	P*tac*	*R. erythropolis* IGTS8	*Pseudomonas putida*	Raheb et al. (2009)
10	P*tipA*	Synthetically synthesized	*R. erythropolis* IGTS8	Dorado-Morales et al. (2021)

The strain *Gordonia alkanivorans* RIPI90A, previously known to be a desulfurizing strain, has special emulsification stabilization properties and was of great interest in the field of biodesulfurization (Shavandi et al., 2009). The *dszABC* genes were cloned under a lac P/O system in pRSG43, thus placing the operon under the control of the well-known lac promoter/operator system, which was evidently sulfate non-repressible. After the plasmid was inserted, the recombinant RIPI90A strain was assayed for desulfurization activity. It showed better activity than the native strain. Although both strains had similar growth kinetics, the recombinant strains had a higher biomass yield. Recombinant RIPI90A also exhibited maximum desulfurization activity at 20 h and produced a higher concentration of the end product of the 4S pathway, which is 2-HBP, while the native strain took 50 h. This publication showed the usage of promoter replacement to improve the efficiency of a biocatalyst (Shavandi et al., 2009).

The *dsz* operon from *Mycobacterium* sp. G3, a naturally desulfurizing organism, was inserted in plasmid pSMT3 downstream of the heat shock promoter, hsp60 and transformed in *Mycobacterium* sp. G3. As a result of this, the recombinant strain was observed to show desulfurization activity by utilizing DBT even in the presence of 0.5 mM sulfate in contrast to the WT strain, where due to the sulfate-repressible WT *dsz* promoter, there was no expression of *dsz* genes (Takada et al., 2005).

In another study, the *dsz* operon from *Rhodococcus* sp. T09 was cloned under the control of the *rrn* promoter in a *Rhodococcus*–*E. coli* shuttle vector. The desulfurization activity was observed even in the presence of 0.4 mM sulfur sources (Matsui et al., 2002).

To overcome the repression due to sulfate, the *dsz* operon from *Arthrobacter* sp. DS-7 was cloned under the constitutive promoter for the neomycin phosphotransferase gene in a plasmid. The desulfurization activity in cured *Arthrobacter* sp. DS-7 harboring the recombinant plasmid was demonstrated even in the presence of sulfate (Serbolisca et al., 1999).

Similarly, the *dsz* operon from *Rhodococcus* sp. FUM94 was cloned downstream to an alkane-responsive P*com8* promoter, and the expression was analyzed in *E. coli* BL21 (DE3). The study demonstrated that the biodesulfurization activity was not repressed in the sulfate medium and also increased with increasing concentrations of DBT (Khosravinia et al., 2018).

Another study used the *dszABC* genes from a naturally desulfurizing organism *R. erythropolis* DS-3 and integrated them into the chromosomes of two strains of *Bacillus subtilis* (Ma et al., 2006). A suitable vector containing the *dszABC* genes ligated to a P*spac* promoter was inserted into the two host strains of *B. subtilis.* Upon integration via homologous recombination, the recombinants lost their amylase activity. Two colonies were further selected, M28 and M29. They were both assayed for DBT desulfurization, and M29 showed better results than M28 and even *R. erythropolis* DS-3 at 36 h of the experiment. M29 was also not inhibited by the end product of the 4S pathway, 2-HBP. Another very important discovery was that strain M29 decreased the interfacial tension in the medium much more than M28 did. This particular difference stems from the two different host *B. subtilis* strains that were used to produce M29 and M28. The decrease in interfacial tension of the medium is due to the production of biosurfactants, which M29 is capable of inducing. Ma et al. noted that this is of particular advantage in an industrial setup. By facilitating the emulsification of the medium by the biosurfactants, the culture increases the access of the organic phase to the desulfurizing cells in the broth (Ma et al., 2006).

A transposon containing a promoter-less reporter gene was constructed and transformed into the *R. erythropolis* KA2-5-1 host cells to detect sulfate, non-repressible promoters that control the promoter-less reporter gene inserted via the transposon (Noda et al., 2002). The construct TnKgfp was prepared, having a promoter-less gene for red shifted green fluorescence protein (rsGFP), the transposon with transposase, and a kanamycin resistance gene. The above transposon was then electroporated into *R. erythropolis* KA2-5-1, and then medium A (having sodium sulfate and sulfate salt of kanamycin) was used to select the recombinants where the transposon (containing the promoter-less reporter gene of rsGFP) gets inserted downstream to a sulfate, non-repressible promoter. Some of the colonies showed fluorescence and were sequenced, following which it was found that three of them had been inserted downstream into ribosomal RNA promoters. However, two of the colonies did not show high homology to any known region. Of these, a colony designated as K1 showed the best fluorescence, indicating the highest promoter strength. An *E. coli* and *R. erythropolis* KA2-5-1 shuttle vector were used to ligate four deletion fragments of the K1 fragment to determine the putative promoter sequence. Sequencing data revealed that the promoter belonged to one *kap1* promoter, which was indeed a sulfate non-repressible promoter.

Following this discovery, the *kap1* promoter sequence was used to clone it in pRDS (having the promoter-less *dsz* operon). It was observed that the WT *dsz* operon containing the transformant was repressed to 1/10th by the presence of sulfate at 0.25 mM, whereas pRKAPDS, where the *dsz* operon was cloned under the control of the *kap1* promoter sequence, showed comparable desulfurization activity as that of the control, and its activity when grown in sulfate was not repressed like in the case of the control.

It was observed that in the presence of 0.25 mM of sulfate, the control showed a decrease in activity, while the pKAPDS showed a distinct increase. Furthermore, at 0.5 mM sulfate, the control showed no activity at all, while 0.14 mmol/g DCW/h activity was detected in the pRKAPDS. As cell growth was also checked in both cultures, it was concluded that the *kap1* promoter sequence not only promotes sulfate non-repressible control on the *dsz* operon but also stimulates cell growth (Noda et al., 2002).

### Metabolic engineering

Two hypotheses were considered regarding the fate of sulfite formed in the 4S pathway. First, the cell converts sulfite to sulfide, which can be further assimilated to biomass; this conversion is done by the enzyme sulfite reductase (SR), and the excess sulfite is converted into extracellular sulfate by the enzyme sulfite oxidoreductase (SOR). Second, SOR converts sulfite to sulfate, and then the cells utilize sulfate to form biomass via SR, thus altering the levels of SR and SOR has a direct effect on the desulfurization activity. The effect of altering SR and SOR was studied using an *in silico* model. Various simulations were carried out, and it was concluded that an increase in the activity of desulfurization was observed when SOR activity increased and SR activity decreased (Aggarwal et al., 2012).

### Enhancing solvent tolerance of the host

Most of the biodesulfurizing strains reported are Gram-positive and exhibit relatively less solvent tolerance. It has been demonstrated that the desulfurization rates were increased when the *dsz* operon from *Rhodococcus* was cloned in *Pseudomonas*, which is known to produce a rhamnolipid biosurfactant. It has been suggested that biosurfactants help in the accessibility of the hydrophobic substrates (McFarland, 1999).

In another study, the *dsz* operon from *R. erythropolis* IGTS8 was inserted into the chromosome of *P. putida* via a vector carrying Mini-Tn5, a transposon. The *P. putida* also had the gene *dszD* inserted into it via a vector pVLT31. The gene fragment for the *dszABC* genes was cloned under a *tac* promoter, which was also sulfate non-repressible. Using the transposon, this fragment was stably inserted into the chromosome of *P. putida*. The desulfurization activity after 18 h of *P. putida* was better than *R. erythropolis* IGTS8. The concentration of 2 HBP was higher in *P. putida* than that of *R. erythropolis* IGTS8. It was noted that *P. putida* is an ideal biocatalyst for the industrial process of biodesulfurization. The organism had an optimum growth temperature of 40^o^C, which is very suitable for an industrial fermentation process. The organism is also known to produce a rhamnolipid biosurfactant, which increases emulsification, thereby increasing the two-step separation process in biodesulfurization (Raheb et al., 2009).

### Inducing biofilm formation as an approach to improved biodesulfurization activity

Recently, Solano et al. used a novel idea of inducing a biofilm formation phenotype in *R. erythropolis.* For this purpose, diguanylate cyclase AdrA from *Salmonella enterica* serovar Enteritidis was expressed, which resulted in enhanced levels of c-di-GMP, which is known to trigger the formation of biofilms (Dorado-Morales et al., 2021). A non-repressible *TipA* promoter was used for expressing the *dsz* operon, and the lac promoter was used for expressing the *dszD* and *adrA* cloned in an operonic configuration. Both the operons were separated by a strong transcriptional terminator from the lambda phage in the same plasmid. The *Rhodococcus* biofilm cells showed very high desulfurization activity compared to planktonic cells (Dorado-Morales et al., 2021).

### Mutagenesis

a) Mutagenizing the upstream region of *dszB*

**FIGURE 4:**
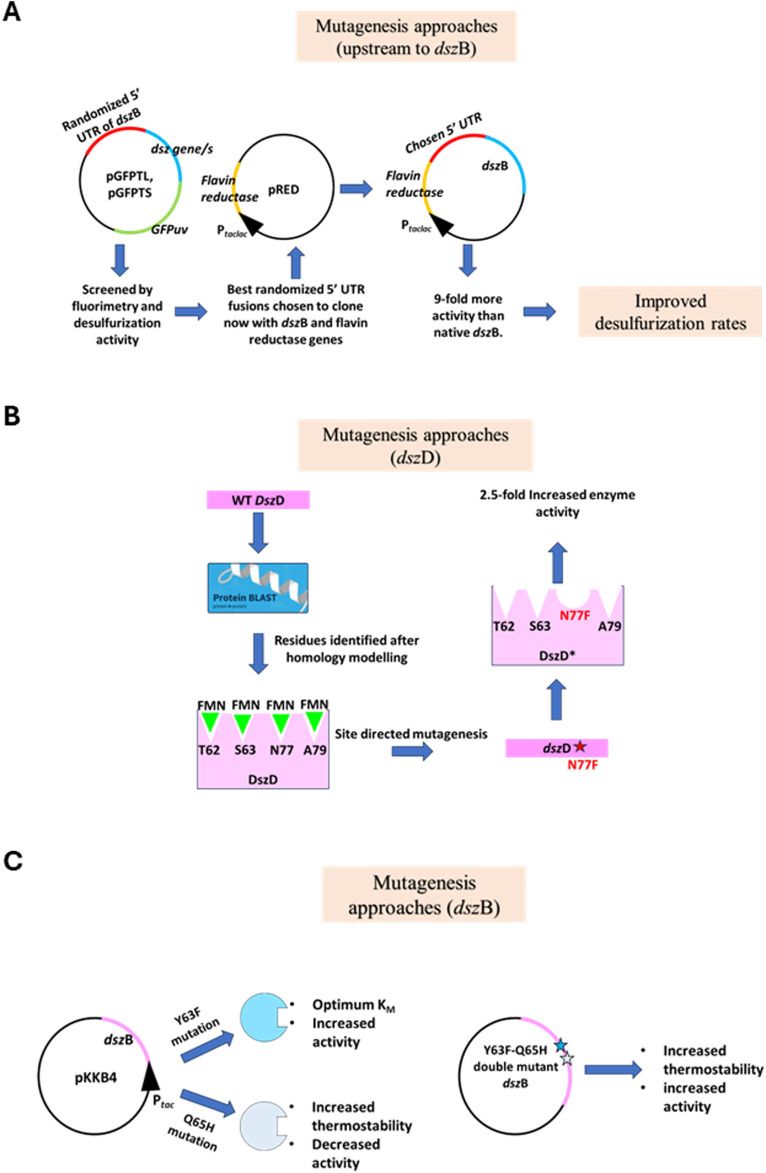
**(A)** A strong 5′UTR of the *dsz*B gene was created. pGFPTL and pGFPTS are translational and transcriptional fusions of GFPuv and a combination of the *dsz* genes, respectively. These plasmids were used to screen the best-performing randomized 5′UTR sequence. The chosen sequence was then used to clone *dsz*B downstream to a flavin reductase gene from *Vibrio harveyi*. The resultant construct yielded 9-fold more activity than the native DszB (adapted from Reichmuth et al. (2000). **(B)** Mutagenesis approaches of DszD enzyme target to increase the specific activity of the enzyme by identifying the FMN-binding sites. Site-directed mutagenesis was carried out at the 77th position, from asparagine to phenylalanine, which increased the specific activity. Adapted from Fallahzedah et al. (2019). **(C)** Site-directed mutagenesis of the *dsz*B gene. A double mutant, having the 63rd position mutated from tyrosine to phenylalanine and the 65th position mutated from glutamine to histidine, yielded increased thermostability and specific activity. Adapted from Ohshiro et al. (2007). The star marks denote mutations incorporated into the sequence.

The rate of desulfurization is limited by DszB, the last enzyme in the 4S pathway, due to its low production, despite the use of a consensus ribosome-binding site. An array of randomized sequences upstream to the start codon of the *dszB* gene was generated (Reichmuth et al., 2004). Transcriptional and translational GFPuv fusions were used with different individual genes of the *dsz* operon or pairs of genes to ascertain the levels of the DszB transcript and protein production with respect to the others in the operon. Mutants were generated using PCR with degenerate primers having mutations in the −17 to −4 regions upstream of the start codon of the *dszB* gene. All of these were used to prepare separate GFP translational fusions. After the appropriate colonies were selected with high DNA content and a high level of GFP fluorescence, DNA was isolated and ligated into the pRED vector, downstream of the flavin reductase gene. A colony named pDSZ/pRED-DSZB-5A had 9-fold more desulfurizing activity than the native *dszB* gene (Figure 4A). This work focused on mutagenizing only the upstream region of the *dszB* gene. Future work can be carried out using other elements, like increasing mRNA stability and increasing the plasmid copy number (Reichmuth et al., 2004).

b) Mutagenesis of DszD

To improve the speed and efficacy of the process, enzymes involved in the process need to have low K_M_ values and broad substrate specificities (Monticello, 2000). Two common types of mutagenesis used are focused and random mutagenesis. Site-directed mutagenesis is one of the methods of focused mutagenesis (Packer and Liu, 2015). Fallahzadeh et al. (2019) worked on the flavin oxidoreductase enzyme, DszD, in the 4S pathway of *R. erythropolis* in order to improve its catalytic efficiency via site-directed mutagenesis. Known to have low catalytic power, DszD is considered the primary limiting factor in the practicalities of biodesulfurization in industries. To ascertain the amino acid residues that majorly contribute to the function of this enzyme, its amino acid sequence was used to identify other homologous molecules with very high similarity using NCBI BLAST (basic local search alignment tool). This showed the key residues in the enzyme to be threonine 62, serine 63, asparagine 77, and alanine 79. These are crucial in binding to the substrate. Site-directed mutagenesis was performed using a single-tube overlap extension (SOEing) PCR, with the WT gene at position 77, replacing the asparagine with phenylalanine (Figure 4B). The oxidoreductase assay revealed a 2.5-fold increase in the catalytic efficiency of the mutant enzyme compared to the wild type (Fallahzadeh et al., 2019). Thus, directed evolution, in this case, focused mutagenesis, has improved the catalytic power of an enzyme, thus paving the avenues for possible research in similar fields on the other enzymes involved in other biodesulfurization pathways.

c) Mutagenesis of DszB

Site-directed mutagenesis was performed on DszB, the 2′-HBPS desulfinase of the *R. erythropolis* strain KA2-5-1 (Ohshiro et al., 2007). The catalytic conversion of 2′-hydroxybiphenyl-2-sulfinate to 2-hydroxybiphenyl is done using the enzyme DszB, and it is considered the rate-limiting step in the 4S pathway. Moreover, the thermostability of the enzyme is also not adequate enough to be appropriate for the industries. Site-directed mutagenesis was carried out at two sites in the WT DszB, the 63rd residue (tyrosine) and the 65th residue (glutamine). The tyrosine residue at the 63rd position was mutated to alanine, lysine, phenylalanine, tryptophan, and serine. The glutamine at the 65th position was mutated to histidine (Ohshiro et al., 2007). The mutated genes, cloned in respective plasmids, were grown in recombinant *E. coli* expression strains, also expressing molecular chaperones GroES/EL to aid folding (Figure 4C).

Among the Y63 mutants, almost all of them showed increased specific activity (units/mg). However, the Q65H mutant showed a decrease in specific activity from the wild type (26.8 units/mg to 21.0 units/mg). However, the optimum temperature and thermostability evidently improved in the mutant Q65H compared to the WT. Although there was a 2–2.5-fold increase in the enzyme activity, the K_M_ value of Y63S increased almost 7-fold compared to the WT, indicating that the substrate affinity decreased in the mutant. Therefore, Y63F was a better candidate for improving DszB because it delivered increased activity without compromising the affinity towards the substrate. As for the Q65H mutant, the optimum temperature was 45°C, a 10-degree increment from the WT’s. Even after heat treatment at 35°C for 30 min, the mutant retained 60% of its activity, while the WT completely lost it. However, as stated above, the K_M_ value was much higher, indicating that the substrate affinity had decreased (Ohshiro et al., 2007).

In order to overcome the drawbacks of the individual mutant enzymes, gene fusion was carried out to give rise to a double mutant, Y63F-Q65H enzyme. The new enzyme showed similar substrate affinity to the WT and showed increased heat tolerance, optimum temperature, and specific activity. The improved performance of the double mutant was even confirmed by transformation into *E. coli* strains, where the strain carrying the mutant showed better desulfurization and thermotolerance (Ohshiro et al., 2007).

d) Directed evolution using a chemostat

A chemostat ensures a continuous culture of cells in large numbers, allowing spontaneous mutations to occur and accumulate in the genotype of the cells, which can give rise to different phenotypes. It is designed to promote these mutations, altering the wild-type phenotype to achieve a desirable outcome. Arensdorf et al. (2002) developed a chemostat to use directed evolution to generate and screen gain-of-function phenotypes in the bacterial population capable of metabolizing a wide variety of sulfur sources other than DBT.

A two-phase sulfur-limited chemostat was designed to select for gain-of-function mutants that have the ability to metabolize many non-DBT sulfur sources. *R. erythropolis* BKO53 was used in the study. The monooxygenases DszA and DszC had different substrate preferences, the ranges of which varied to a great deal among the three broad categories of sulfur sources, viz., benzothiophenes, thiopenes, and di-aryl and aryl alkyl sulfides. In this sulfur-limited chemostat, the selective pressure was altered after consecutive mutations in the dominant culture. A single colony was isolated, which could metabolize both octyl sulfide and 5-methyl benzothiophene (5-MBT). The gene *dsz*A in the colony had three nucleotide mutations, which were responsible for the octyl sulfide gain of function. A single-amino acid change, from valine to phenylalanine, due to a transversion in the 261^st^ codon of the *dsz*C gene resulted in the gain-of-function mutation of 5-MBT metabolism. Following this, the random chimeragenesis on transient templates (RACHITT) technique was used to generate *dsz*C clones with codon 261 mutations, which were further transformed into a *dsz*- *R. erythropolis* JB55, and the clones were assayed for activity. Although more improved variants were not obtained, some of them lost all activity, highlighting the importance of codon 261 in functionality. This work showed that a chemostat is a very effective way to broaden the substrate preferences of desulfurizing strains, which will be advantageous in the commercial expansion of biodesulfurization (Arensdorf et al., 2002).

e) RACHITT for improving the activity of DszC

In DNA shuffling, double-stranded DNA of homologous gene families is fragmented randomly by DNase I, followed by recombination of the fragments created by fractionation of the parent genes. This creates a diverse library of gene families that are genetically unique and different from the parent sequences as well (Pelletier, 2001). Repeated cycles of shuffling create gene products with better substrate affinity and specificity, better activity, and improved folding of the protein (Crameri et al., 1998). However, one disadvantage of this method is that when it is applied to areas of the gene having low sequence homology, the recombination event is relatively inefficient, leading to the formation of a small number of variants. To overcome this, a novel strategy called RACHITT was developed that proved to yield better recombination frequencies (Coco et al., 2001). In RACHITT, instead of double-stranded DNA being fragmented, single-stranded parental DNA is fragmented and then hybridized into another single-stranded DNA, which is termed the “scaffold.” The scaffold serves two main purposes: first, it ensures the incorporation of uracil, making it easier to degrade it later and eliminate unshuffled parental DNA sequences that have failed to undergo any recombination, and second, by ensuring that the scaffold is homogenous throughout, biases in hybridization are minimized. A novel aspect of this process is the “overhang-trimming,” where the non-hybridized sequence is trimmed to generate very small sequences, which was not achievable with other methods (Pelletier, 2001).

**FIGURE 5:**
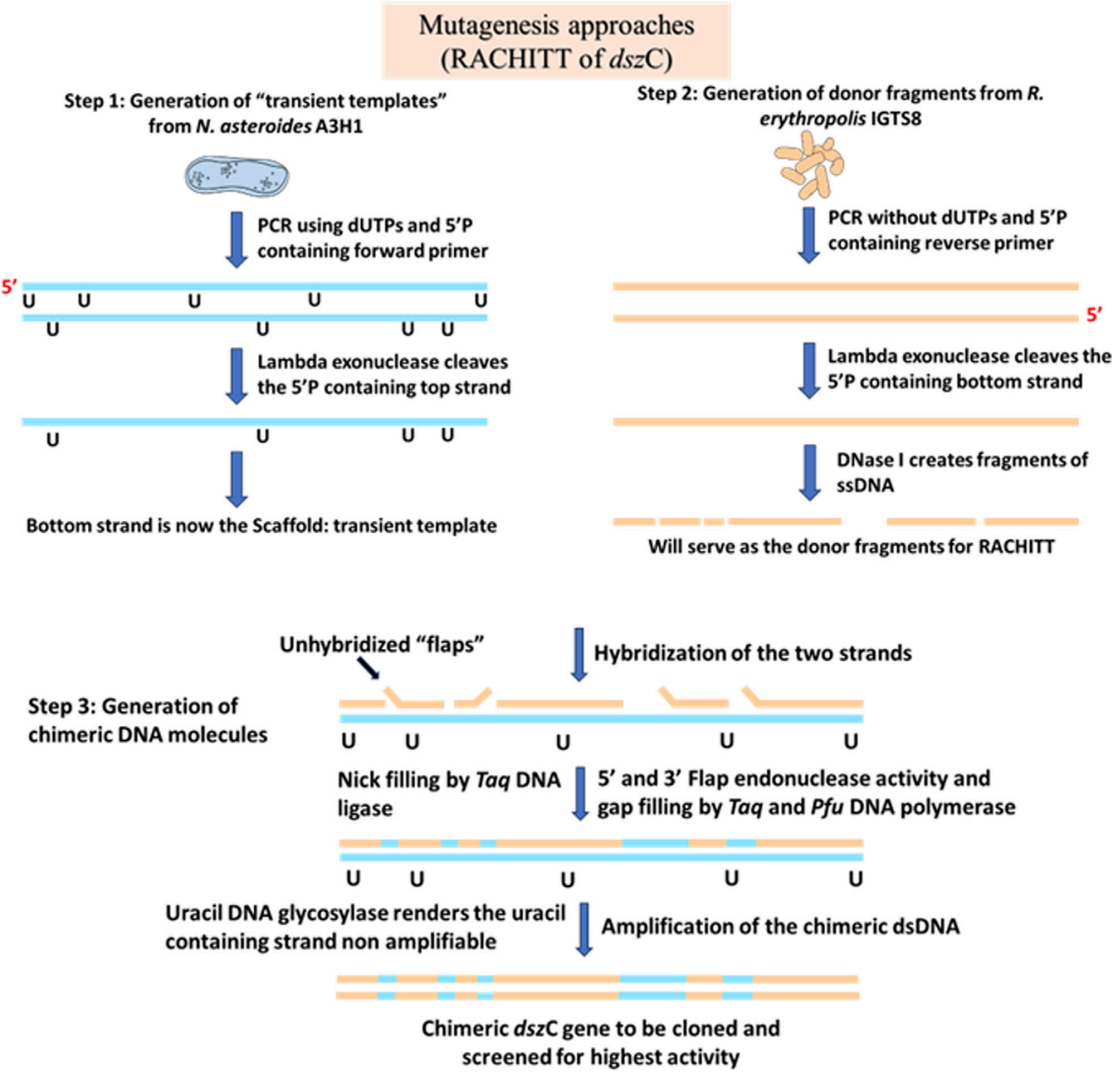
Schematic diagram showing the steps of a RACHITT protocol. In step 1, the transient templates are created. During amplification, dUTPs are incorporated to make the strands further vulnerable to UDG attack in the later steps, and the forward primer has a 5′ phosphate to make it vulnerable to the Lambda exonuclease attack to create a single-stranded DNA molecule. In step 2, the donor fragments are prepared following a similar protocol, except the incorporation of uracil in the PCR amplification step. In step 3 of RACHITT, the actual chimeric dsDNA molecule is created. The unhybridized, or non-annealed sequences, or “flaps” are digested by Flap endonuclease activity of Taq DNA polymerase, followed by the filling of gap regions and sealing of nicks by Taq DNA ligase. The uracil residues in the transient template scaffold molecule make it vulnerable to uracil DNA glycosylase, and the molecule can no longer be amplified by PCR. The true chimeric molecules may then be amplified, cloned, and screened for higher activity. Adapted from Coco et al. (2001), Coco (2003).

This technique was used to develop an improved biodesulfurization strategy by engineering the dibenzothiophene monooxygenase enzyme (DszC) from two homologous parent genes from *Rhodococcus erythropolis* IGTS8 and *Nocardia asteroids* A3H1 (Coco et al., 2001). It was noted that RACHITT yielded better diversity and improved chimeric properties than those obtained from random mutagenesis of target genes. DszC from IGTS8 had a better specific reaction rate for less alkylated derivatives of DBT, whereas that from A3H1 had a higher substrate affinity for complex alkyl derivatives of DBT. The genes were 89.9% identical. After the random crossover event between these two parent sequences of *dsz*C from IGTS8 and A3H1, the resultant library was cloned and transformed into a non-desulfurizing *Rhodococcus* (Figure 5). The colonies were then screened on an agar plate containing diesel and DBT. Restriction fragment length polymorphism (RFLP) was used to analyze the chimeras, which revealed spontaneous mutations accumulated in the recombinants along with the crossover products (Coco et al., 2001).

Out of six selected clones, four had significantly greater activities than both of the parental clones. To enhance the activities, affinities, and extent of sulfur oxidation of the created clones, they were grown in oil containing low sulfur content. About 109 such clones were obtained after screening, proving that the chimeric clones had better affinities than their parental genes. One such clone had the best properties of the parental genes, including higher activity (higher rate of reaction) and improved extent of sulfur oxidation, as observed using shake-flask assays. The agar plates containing the chimeric library colonies were exposed to indole vapors, and a 20-fold increase in indigo production (conversion of indole to indigo) was observed (Coco et al., 2001).

f) Mutagenesis for improving feedback and substrate inhibition of DszC

**FIGURE 6:**
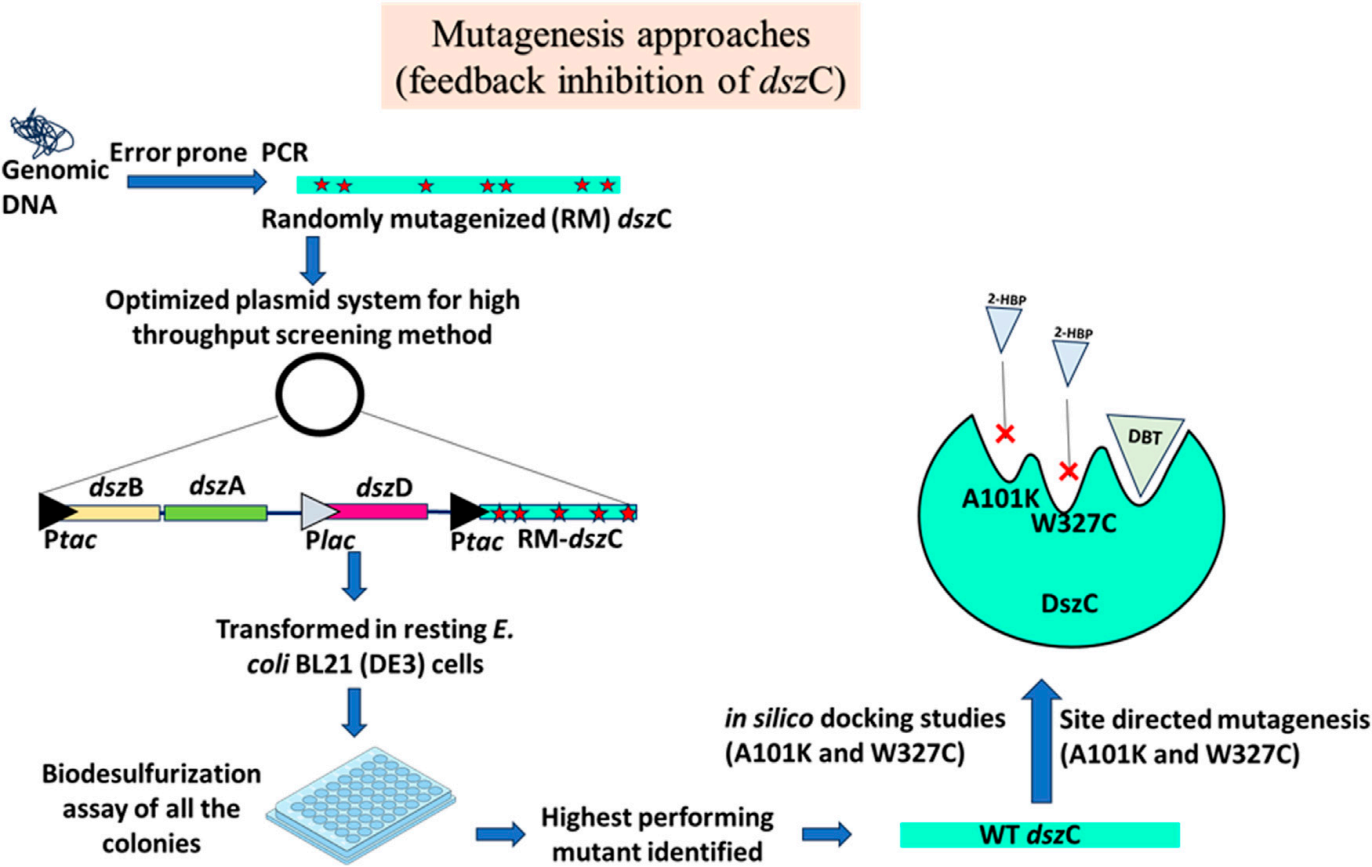
Mutagenesis approaches of the DszC enzyme targeting to reduce the 2-HBP-mediated feedback inhibition. An optimized plasmid system, containing an elaborate heterologous promoter system (P*tac*/P*lac*) and genetic rearrangement, was developed to maximize biodesulfurization activity in which the *dsz*C gene was subjected to random mutagenesis. The resulting clones were screened, and the highest performing mutant was identified. Site-directed mutagenesis was carried out, at the 101th and 327th positions, which helped reduce the enzyme’s feedback inhibition. Adapted from Li et al. (2019). The star marks denote mutations incorporated into the sequence.

Li et al. (2019) targeted the feedback inhibition by HBP on DszC. HBP is a noncompetitive inhibitor of the enzyme, implying that its binding site on DszC is different from that of the substrate, DBT. There are one or more binding sites on DszC for HBP, but not all of them have been properly identified. So, a directed evolution approach encompassing protein engineering, combinatorial mutagenesis, and a well-established high-throughput screening method was used to develop a double mutant of the DszC mutant AKWC (A101K/W327C). The increase in the IC_50_ value indicated that the feedback inhibition of the AKWC mutant has been substantially reduced after mutating these two residues (Figure 6). The group declared that they successfully used desensitization engineering coupled with the overexpression of the desensitized enzyme to remove the feedback inhibition bottleneck of the 4S pathway (Li et al., 2019).

Next, the same group attempted to ameliorate the substrate inhibition problem of the same enzyme, DszC (Li et al., 2020). They experimented with a mutant of the DszC enzyme from *R. erythropolis*, DszC AKWC (mentioned above). The enzyme usually exists as a 4-mer or an 8-mer, and the dimeric interface of the structure was mapped to demonstrate 40 amino acids associated with the binding of the two known substrates of the DszC, DBT and FMN. Alanine scanning was performed on this dimeric interface of the enzyme, and subsequently, the 413th amino acid was mutated to a variety of amino acids, a mixture of polar and nonpolar, and the activities were checked using two different concentrations of DBT (1.5 µM and 1 mM). The one where the 413th amino acid was mutated to isoleucine retained 57% activity with respect to the wild type, even in the higher (1 mM) DBT concentration. It was reported that the K_I_ was 5.6 times higher than that of DszC AKWC. The enzyme activities of DszC, DszC AKWC, and DszC AKWCPI were measured in different concentrations of HBP and DBT. It showed that the AKWCPI mutant retained almost 56% activity with respect to the WT enzyme, even in 20 µM HBP. This was the first study conducted aiming to tackle the problem of substrate and feedback inhibition of the DszC enzyme in the 4S pathway (Li et al., 2020).

In 2023, a group worked with the DszC enzyme of *R. erythropolis* IGTS8 again, which is most vulnerable to feedback inhibition and substrate inhibition (Neves et al., 2023). The enzyme is inhibited by the product, 2-HBP, as well as an intermediate of the pathway, HBPS. The authors used both molecular docking and molecular dynamic simulation methods to determine four potential binding sites through which HBPS and 2-HBP may inhibit the enzyme. This study followed thorough work that investigated the mechanistic details of the catalysis of both DszC and DszB enzymes, which even included the discussion of the residues in these enzymes that might be targets for site-directed mutagenesis (Barbosa et al., 2018; Sousa et al., 2020). They stated that their findings on rational designing of these enzymes, while used in conjunction with directed evolution strategies, will lead to a better engineered enzyme.

### Removal of overlap

**FIGURE 7:**
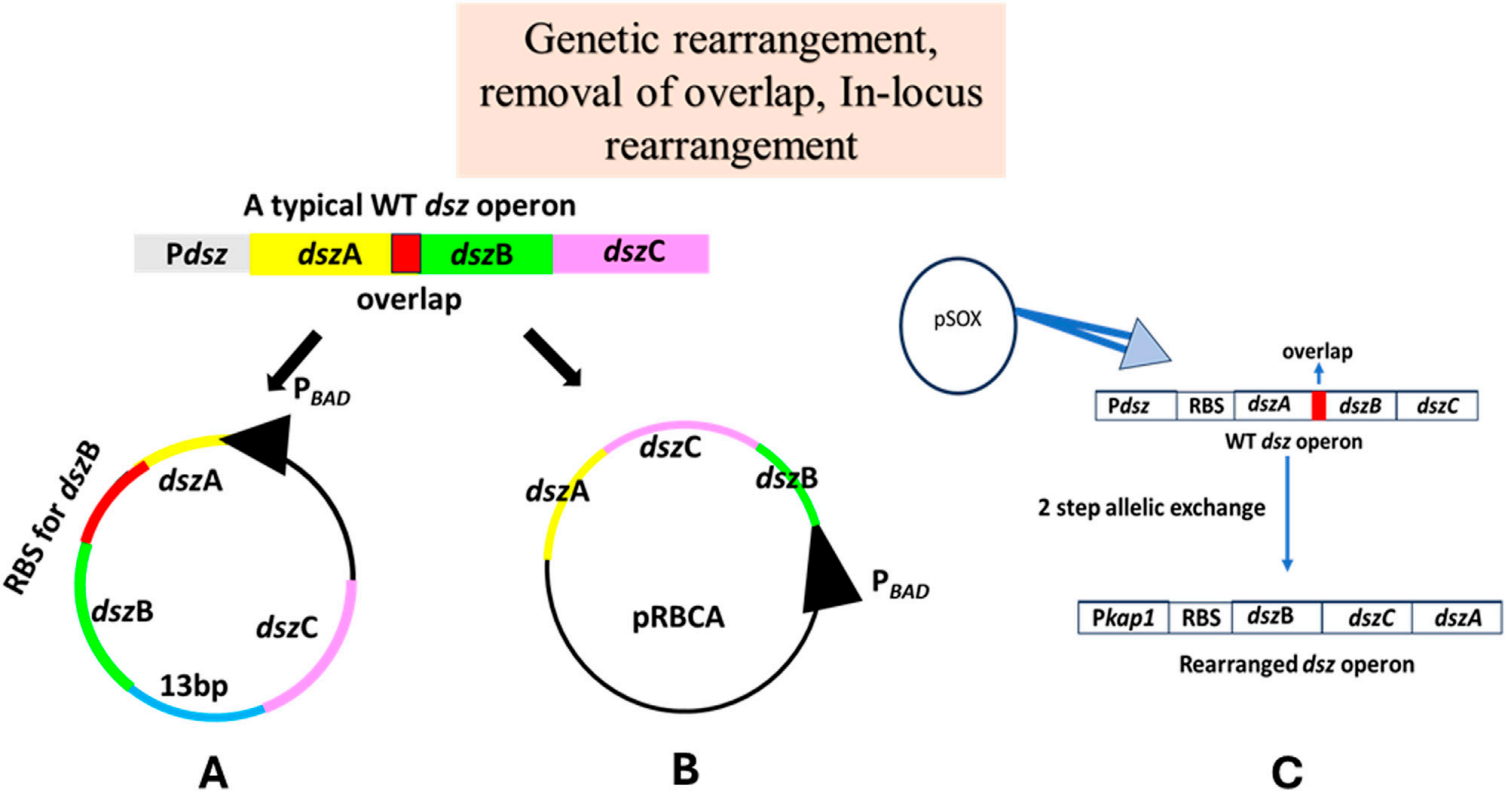
Schematic diagram demonstrating the various genetic engineering strategies carried out to increase the rates of the 4S pathway: **(A)** overlap removal: to increase the transcription and translation rates of the *dsz*B gene in the operon, a separate RBS was introduced upstream to *dsz*B [adapted from Li et al. (2007)]; **(B)** genetic rearrangement: overlap extension PCR was used to reconstruct and rearrange the *dsz* operon to *dsz*BCA [adapted from Li et al. (2008)]; **(C)** *in locus* genomic rearrangement of the *dsz* operon: to increase the rates of transcription of the *dsz*B gene, it was cloned under a sulfate non-repressible promoter [adapted from Martzoukou et al. (2013)]. The genes are not drawn to scale.

The quantitative real-time PCR of the total mRNA obtained from *R. erythropolis* DS-3 was performed with gene-specific primers against all three genes of the operon, *dsz*A, *dsz*B, and *dsz*C, and the mRNA level ratio was found to be 11:3.3:1 (Li et al., 2007). However, in an unexpected observation, Western blot analysis of the three individual proteins showed that DszA is present in the highest amount, followed by DszC and then DszB, highlighting the probable inefficient translation of DszB. Thus, a gene analysis was conducted to see the topology of the genes in the operon, which revealed that there was a 13 bp gap that exists between the 3′ end of the *dsz*B gene and the 5′ end of the *dsz*C gene in WT *dsz* operon and an overlap between the termination codon of *dsz*A and the initiation codon of *dsz*B. By carrying out overlap extension PCR of the genes and upstream and downstream regions of the native *dsz* operon, they reconstructed the *dsz* operon removing the overlap, where the *dsz*B gene was provided with a different and separate Shine–Dalgarno ribosome-binding site (Figure 7A). Li et al. (2007) worked with primarily two strains, *R. erythropolis* DS-3, a strain capable of desulfurizing DBT and its derivatives, and *R. erythropolis* 4.1491, which did not harbor the *dsz* operon (Li et al., 2007). The latter was electroporated with the native *dsz* operon-containing plasmid called DR-1 and the one containing the redesigned operon designated as *R. erythropolis* DR-2. Quantitative real-time PCR analysis of all three genes in all three strains showed no significant difference between the *R. erythropolis* DS-3 and the DR-1 and DR-2 strains. However, Western blot analysis revealed that DszB levels were higher in DR-2 than in DR-1. In biodesulfurization activity assays, DR-2 was visibly better during the first 3 h of analysis, and 96% conversion was achieved during the first 5 h by this strain. The maximum rate was 120 μmole/g (dry weight of cells)/h for DR-2, while it was only a meager 26 μmole/g/h for DR-1. These sets of experiments conducted proved that redesigning the native *dsz* operon and removing the 13-bp gap between genes *dszB* and *dszC* were instrumental in achieving a considerable increase in the rates of desulfurization (Li et al., 2007).

### Genetic rearrangement

In order to make the process of biological desulfurization an industrially suitable method, the catalytic rate of the enzymes has to be high, approximately 1.2–3 mMol of DBT/g (dry weight of cells)/h (Li et al., 2008). Owing to the fact that prokaryotic transcription and translation are coupled, the rate of transcription and the number of mRNA transcribed per gene depend on the distance between the gene and the promoter of the operon. This is called polar transcription, and Li et al. worked on genetically rearranging the genes in the operon to tackle this problem. Real-time quantitative PCR of all genes revealed that the transcription levels were indeed not similar to each other, based on the threshold cycle (C_T_) values of each gene (11:3.3:1 for *dsz*A, *dsz*B, and *dsz*C, respectively). This was in consistent with their position relative to the promoter in the operon. Overlap extension PCR with gene-specific primers was carried out by the group to create the rearranged operon, *dsz*BCA. This particular rearrangement was done to remove the constraint put by the last enzyme in the pathway, DszB, which is the rate-limiting step owing to its low expression level and low catalytic activity. A shuttle vector was used to express both the reconstructed and native *dsz* operons in *R. erythropolis* CGMCC 4.1491, a strain that is incapable of desulfurization by itself (Figure 7B). The culture that was transformed with the native *dsz* operon was designated as *R. erythropolis* DRA, and the one that obtained the rearranged operon was called *R. erythropolis* DRB. It was found that *R. erythropolis* DRB had much higher levels of DszB and DszC mRNAs than *R. erythropolis* DS-3, a desulfurizing strain found in soil (mRNA of *dsz*A:*dsz*B:*dsz*C was 1:16:5 after the rearrangement). Western blot analysis followed similar trends as did the qRT-PCR assays. In the biodesulfurization experiments, although end products of the 4S desulfurization pathway, 2-HBP, were detected in all three strains, only the DS-3 and the DRA strains showed the presence of intermediate products as well. When the rate of desulfurization was assayed, the DRB strain performed much better as 96% of the DBT was desulfurized within 3 h and the maximum rate achieved by the culture was 32 μmol of DBT/g (dry weight)/h in contrast to only 26 μmol of DBT/g (dry weight)/h as observed in the DRA strain. This proved that the DRB strain (housing the rearranged *dsz* operon) is much more capable of desulfurization than the DRA strain, which housed the native *dsz* operon (Li et al., 2008).

### An *in locus* combinatorial approach

In 2023, a group worked with an engineered strain of *R. qingshengii* IGTS8, where they performed an *in locus* homologous recombination to give rise to an 80-fold increase in biodesulfurization compared to the wild type (Martzoukou et al., 2023). This was achieved using all the genetic manipulation knowledge about using sulfate non-repressible promoter P_*kap1*_ removing overlap between *dsz*A and *dsz*B genes, as well as genetic rearrangement of the usual *dsz*ABC to a rearranged *dsz*BCA operon (Li et al., 2008; Noda et al., 2002). This model biocatalyst can be maintained without the need for antibiotic selection. They carried out several comparative studies between these two strains, P_*kap1*_ *dsz*BCA and P_*kap1*_ *dsz*ABC. These two strains and the wild type showed similar profiles in time-dependent biomass growth. In terms of biodesulfurization using DBT as a sole sulfur source analyzed by the production of 2-HBP (µM), the rearranged operon outperformed the P_*kap1*_ *dszA*BC operon. Next, the researchers investigated the effects of the *in locus* rearrangement in the case of biodesulfurization activities with different inorganic and organic sulfur sources. DMSO was the organic sulfur source in which the strains showed the maximum activity (units/mg of dry cell weight). However, in the presence of cysteine and methionine, although the overall biodesulfurization activity was greatly reduced for all the strains, the recombineered strains showed a 10-fold increase in the case of methionine and up to 40-fold in the case of cysteine as the sole sulfur source (Figure 7C). Given that genome engineering tools for *Rhodococcus* are vastly limited, this study could prove pivotal for other such work in the future to create strains with enhanced bioremediation (biodesulfurization) abilities (Liang and Yu, 2021; Martzoukou et al., 2023).

### Surface display and permeabilization of the cell membrane to overcome mass transfer limitation

**FIGURE 8:**
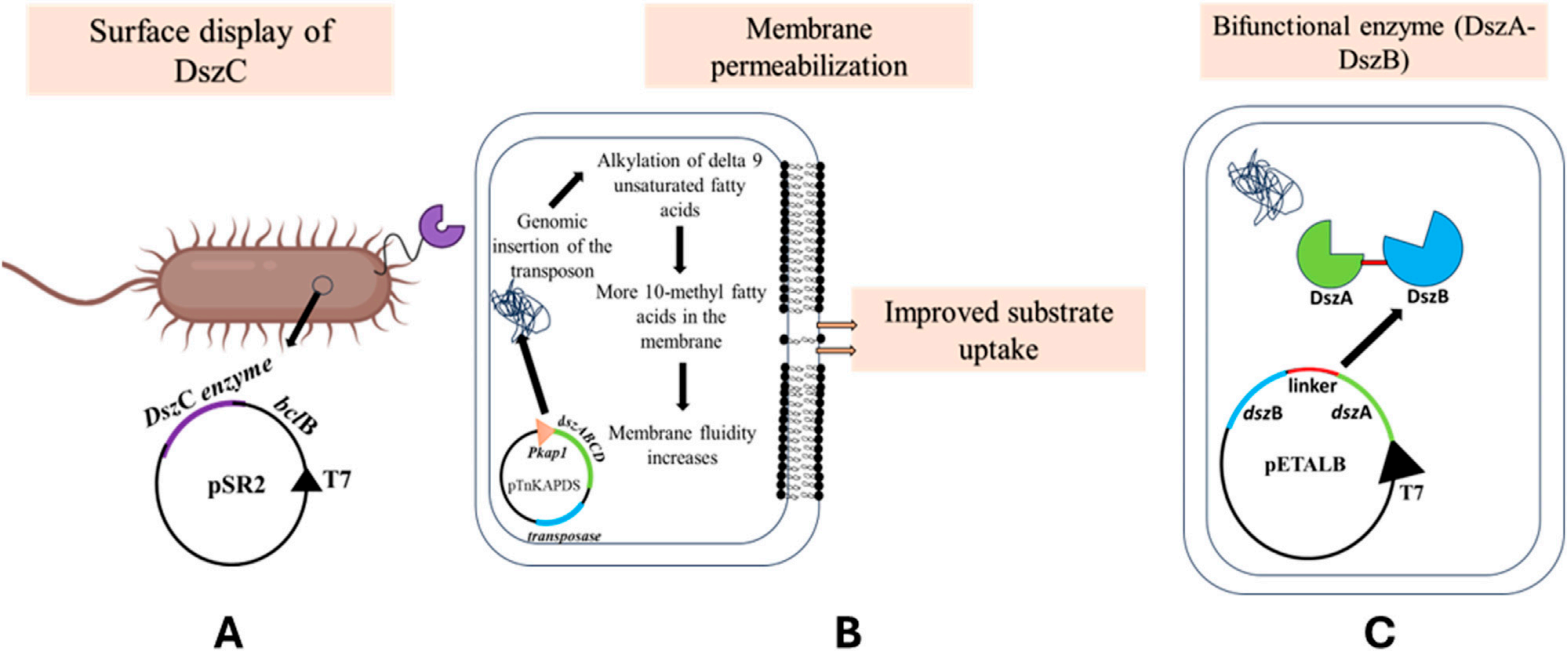
**(A)** Utilizing the *bcl*B signal sequence from *Bacillus anthracis* Sterne*,* the pSR2 plasmid was constructed, which overexpressed the translational fusion of *dsz*C and *bcl*B, under the T7 promoter. The recombinant *E. coli* BL21 (DE3) cells displayed the DszC enzyme on the cell surface. This reduced the mass transfer limitation faced by the cells during the uptake of DBT into the cells [adapted from Rangra et al. (2018)]. **(B)** This is another method of reducing the mass transfer limitation during the uptake of DBT inside the cells. The reconstituted *dsz* operon, containing *dsz*A, *dsz*B, *dsz*C, and *dsz*D genes, was cloned under the P*kap1* promoter along with a transposon. The cells showing higher desulfurization activity were assessed to have a higher percentage of alkylated unsaturated fatty acids in the membrane, thus increasing its fluidity and possibly incrementing substrate uptake [adapted from Watanabe et al. (2003)]. **(C)** Co-localizing enzymes DszA and DszB using a linker, under the T7 promoter, produced a bifunctional enzyme, yielding 16-fold higher desulfurization rates (adapted from Indian patent “Recombinant vector for biodesulfurization and uses thereof” no. 386539).

A major limitation to using biodesulfurization via the 4S pathway on an industrial scale is the one posed by mass transfer limitation. In our laboratory, a well-established technique of microbial surface display was used to display the monooxygenase, DszC, on the cell surface of *E. coli*. This study used one such anchoring domain, BclB from *Bacillus anthracis* Sterne to express DszC on the surface *of E. coli* (Figure 8A) (Rangra et al., 2018).

A desulfurizing strain *Gordonia* sp. IITR100 was used to obtain the gene for *dsz*C, and *B. anthracis* Sterne was used to obtain the gene for BclB. Of the vectors that were constructed, pSR1 had only the gene of *dsz*C cloned in it, pSR2 had *bcl*B cloned upstream to *dszC* in pSR1, pSR3 had only *bcl*B cloned in pET29a, pSR4 was constructed with *bcl*A gene from *B. anthracis* Sterne in pET29a, and pSR5 had *bcl*A cloned downstream to *dszC*.

The expression of proteins was confirmed by SDS-PAGE. It was observed that the amount of surface-displayed DszC was more than the intracellular amount of the same enzyme. Moreover, BclB was a better choice as an anchoring motif for displaying DszC than BclA. Scanning electron microscopy and transmission electron microscopy studies confirmed that in the case of the pSR1 transformant, the cell surface appeared smooth, but the pSR2 clone showed rough surfaces. DBT conversion to DBT sulfone was higher in the case of surface-displayed DszC than intracellular DszC.

Jose and von Schwichow (2004) noted that to facilitate the surface display of such enzymes, cofactor regenerating enzymes may also be displayed alongside them (Jose and von Schwichow, 2004). The advantage of this technique lies in its ability to decrease the time the fuel gets exposed to the displayed enzyme, resulting in a multi-fold increase in the reaction rate (Rangra et al., 2018).

It was hypothesized that the transport of the substrate, i.e., DBT from the oil phase in the culture to the cell, is a rate-limiting step, so if the transport is enhanced, there might be an increase in desulfurizing activity (Watanabe et al., 2003). A transposon, TnKAPDS, having the *dsz* genes under the control of *kap1*, which was previously shown to be a sulfur non-repressible promoter, was created (Noda et al., 2002). It also housed a kanamycin resistance gene and transposase enzyme coded on either side. This construct was electroporated into *R. erythropolis* MC1109, and the culture was grown in a medium containing 1 mM DBT (Figure 8B). The host was originally incapable of metabolizing DBT, so only the recombinants will survive. This way, two strains were selected, MC0122 and MC0203.

It was found that the MC0203 strain had twice as much desulfurization activity compared to MC0122 and even *R. erythropolis* KA2-1-5, which is a naturally desulfurizing strain.

The MC0203 strain was further used to assay the metabolism of DBT and 4,6-diethyl DBT. In both cases, it was noted that the rates of degradation were higher at high temperatures than at lower temperatures. In addition to that, they observed that both the substrates were completely converted to the 2-HBP and 3,3-diethyl-2-hydroxybiphenyl, respectively. Composition analysis of the fatty acids of both strains was then carried out. The group observed that MC0203 had 28%–41% more of 10 methyl fatty acids than MC1109. It was hypothesized that the insertion of the transposon in the genomic DNA in the host might have caused the alkylation or methylation of delta 9 unsaturated fatty acids. The inference was that this might have led to making the cell membrane more fluid, which ultimately led to enhanced substrate uptake and, thus, increased biodesulfurization. This work must be followed up with analysis and sequencing of the part in the chromosome where the transposon got inserted to understand the mechanism of enhanced biodesulfurization better and hopefully design more efficient techniques to carry on the motive of increasing rates of desulfurization (Watanabe et al., 2003).

### Construction of a bifunctional enzyme

The enzyme DszB has been reported to be the rate-limiting enzyme, and the DszB levels are comparatively low in the cell. In our laboratory, we constructed a bifunctional enzyme by fusing DszA and DszB using a flexible linker (Figure 8C). The promoter region had only 52.5% homology. Li et al. (1996)). The bacterial cell harboring the bifunctional enzyme resulted in 16-fold higher activity than in the cell where the enzymes were individually expressed (Agrawal et al., 2022, Indian Patent, recombinant vector for biodesulfurization and uses thereof, no. 386539).

### Regulating the expression of genes

The regulatory mechanism of the *dsz* operon in any native desulfurizing bacteria had not been previously elaborated. A thorough analysis of the promoter region of the *dsz* operon in *Gordonia alkanivorans* RIPI90A was carried out, and the promoter region was compared with that of *R. erythropolis* IGTS8 (Shavandi et al., 2010). The promoter region had only 52.5% homology. Li et al. (1996) investigated the topology of the upstream region of the *dsz* operon and found out two possible regulatory sequences by hydrazine mutagenesis and cloning the region upstream to the *lacZ* reporter gene in *E. coli.* Carrying out an S1 nuclease protection assay, the promoter was discovered to be starting from the −46 position upstream to the *dszA* initiation codon. Transcription is repressed by sulfates and cysteine. A region from −146 to −121 was also elucidated by deletion analysis, which plays the role of an enhancer to the operon promoter, hypothesizing that multiple transcriptional activators and repressors might bind to this region, regulating the operon expression (Li et al., 1996). An *in vitro* pull-down assay to determine the proteins responsible for regulating the expression of the operon in *Gordonia* sp. IITR100 was carried out (Murarka et al., 2019). A protein identified as a TetR family protein (Protein ID: WP_010840674.1) was found to serve as an activator of the operon. About 3.6-fold increase in biodesulfurization activity was observed when the TetR family protein supplied from a plasmid was expressed using sub-optimal inducer concentrations. Thus, supplying a transcriptional activator like TetR can also be used as a strategy to enhance the biodesulfurization activity of cells. Further research needs to be conducted on the regulatory mechanisms of the *dsz* operon undertaken by the protein before implementing it industrially on a much larger scale.

Following the discovery of the role of TetR as a transcriptional activator in the process, we also found WhiB1 (a member of the WhiB family of transcriptional regulators) as another protein binding to the promoter region of the *dsz* operon, discovered through an *in vivo* assay. WhiB1 was found to be regulating the expression of *dsz* operons in its own host *Gordonia* sp. IITR100 and the recombinant host *R. erythropolis* IGTS8 (Murarka et al., 2019; Murarka et al., 2020). This regulation led to a 40% decrease in desulfurization, as ascertained by Gibbs assay, inferring that the protein may play an inhibitory role in *dsz* operon expression. Along with this, all the strains expressing the protein showed decreased cellular growth and significant alterations in the cellular shape. The binding of WhiB1 to the promoter region was confirmed by an electrophoretic mobility shift assay (EMSA). WhiB1 has a repressive effect on the *dsz*C gene, which was thought to be due to the direct repression of the operon and inhibition of its transcriptional activator, TetR. A point mutation (Q116A) in the DNA-binding region resulted in a decrease in repression, further confirming that repression is mediated by binding to the promoter region of the *dsz* operon. This repression of the *dsz* operon can also be a very potent target, which ought to be explored to improve biodesulfurization in these strains (Murarka et al., 2020).

Functional metagenomics was employed to identify a functional desulfurization operon in a fosmid capable of rendering biodesulfurizing abilities to the host (Martín-Cabello et al., 2020). A clone out of 185,000 others was identified, which was capable of indigo production, named UPO21 (Terrón-González et al., 2016). This particular clone also had a *dsz* operon where the gene products of the three overlapped genes, monooxygenases, *dsz*A and *dsz*C, and the desulfinase, *dsz*B, were found to be very similar (∼80%) to the enzymes found in the previously characterized organisms. However, a different flavin oxidoreductase gene was also observed to be very closely linked with the other three and had very little similarity (52%), and this was designated as *dsz*E. This was the first case where the desulfurizing operon was found to be in proximity to an oxidoreductase gene. Along with *dsz*E, a transcriptional regulator gene, *dsz*R, was discovered to be in the clone. Through BLAST, DszR was found to be a σ^N^-dependent activator that binds to two contiguous sequences upstream of the promoter region. This feature is completely different from the *dsz* operons of *R. erythropolis* IGTS8 and *Gordonia* spp., where their promoters are σ^70^-dependent. DszR is involved in bringing about a unique repression mechanism of the *dsz* operon, where DszR being transcribed from a heterologous promoter would render the σ^N^-dependent promoter region ineffective, or an “insulator,” thus effectively stopping the transcription of the *dsz*E gene. The other genes in the operon, being translationally coupled, will consequently also be repressed. Due to it not being sulfate-repressible, this system can be exploited for large-scale desulfurization (Martín-Cabello et al., 2020).

A 2024 study investigated the number and nature of metabolites like 11Z-eicosaenoyl-EA, carboxyethyl isoleucine, taurine, 2-hydroxynicotinic acid, and nicotinic acid created in the cellular environment through the 4S desulfurization pathway of *R. erythropolis* in response to the substrate DBT and varying concentrations of the product 2-HBP. The levels of the metabolites were examined in the presence of DBT and increasing concentrations of 2-HBP, and their possible roles in the desulfurization process were discussed. This metabolomics approach is expected to be instrumental in making the process more adaptable for industrial applications (Ahmad et al., 2024).

## Conclusion and future prospects

More than two decades have been dedicated to researching ways to improve biodesulfurization, but an industrial process for its implementation has not yet been established (Mohebali and Ball, 2016). In addition to the bioreactor design, the choice of microorganisms is also a ruling factor. Most of the genetic engineering approaches reported to date have been carried out in isolation (Figures 2–8). They resulted in improved activities, but there is a need to combine multiple such engineering approaches within one strain without compromising the cellular physiology or causing metabolic burden. With the advent of newer and faster genome engineering methods, the recombinant gene cassettes can be integrated into the genome. This will overcome the plasmid instability issue, which is a major problem when the process is carried out in a bioreactor. The advantage of using one strain vs. a mixture of strains is that the purity of the strain and the loss of strain can be frequently monitored.

**FIGURE 9:**
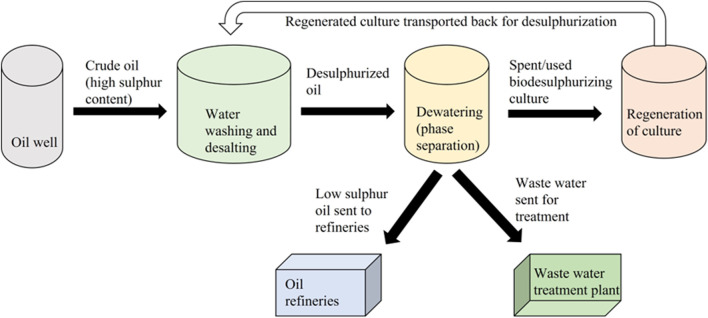
Proposed scheme for implementing the biodesulfurization technology in an industrial setup [adapted from Monticello (2000)].

Although deep desulfurization processes currently exist that can reduce the sulfur level to 15 ppm, biodesulfurization offers significant potential for several reasons: 1) it can be used for heavy-crude oil samples, which are seven times more abundant than the light conventional crude oil samples (Figure 9). The process can be applied during the oil washing step; 2) it can also result in the reduction of the viscosity of heavy crude oil, which will further make this amenable to refining; 3) it can reduce pipeline corrosion; 4) it can be used for treating heavier fractions of fuel; and 5) isolation of newer naturally occurring strains with improved activity will have potential in bioremediation as they can result in the biotransformation of larger molecules into smaller ones, which can be easily degraded by the native microbial community.

To date, only DBT and a few other thiophenic compounds have been targeted, but the emerging need is to focus on sulfur removal from larger structures such as asphaltenes.

Thus, ample research needs to be conducted to investigate the host factors and engineer better biocatalysts with a broader substrate range, enhanced thermotolerance, and higher specific activities. The highest reported increase in biodesulfurization in engineered strains is 80-fold compared to the wild type (Martzoukou et al., 2023), while the desired rate for a biocatalyst to achieve biodesulfurization in industries is a 500-fold increase (Kilbane II, 2006). Although there is still a long road to actualizing this goal, biodesulfurization can be a practically applicable reality.
